# Hippo signaling pathway in companion animal diseases, an under investigated signaling cascade

**DOI:** 10.1080/01652176.2021.1923085

**Published:** 2021-05-14

**Authors:** Shaydee J. Budel, Marloes M. Penning, Louis C. Penning

**Affiliations:** Department of Clinical Sciences, Faculty of Veterinary Medicine, Utrecht University, Utrecht, The Netherlands

**Keywords:** Equine, canine, feline, Hippo pathway, YAP/TAZ signaling, liver, mammary gland, verteporfin

## Abstract

The Hippo pathway is a highly conserved kinase cascade in mammals with the proteins YAP and TAZ as its most important downstream effectors that shuttle between cytoplasma and nucleus. It has a crucial role in processes such as embryogenesis, organ size control, homeostasis and tissue regeneration, where mechanosensing and/or cell-cell interactions are involved. As the pathway is associated with many essential functions in the body, its dysregulation is related to many diseases. In contrast to human pathology, a PubMed-search on Hippo, YAP/TAZ and companion animals (horse, equine, dog, canine, cat, feline) retrieved few publications. Because of its high level of functional conservation, it is anticipated that also in veterinary sciences aberrant Hippo YAP/TAZ signaling would be implicated in animal pathologies. Publications on Hippo YAP/TAZ in companion animals are mainly in cats and dogs and related to oncology. Here, we emphasize the important role of YAP/TAZ in liver diseases. First the liver has a remarkable regeneration capacity and a strict size control and the liver has a moderate liver cell renewal (homeostasis). The last years numerous papers show the importance of YAP/TAZ in hepatocellular carcinoma (HCC), hepatocyte differentiation and bile duct epithelial (BEC) cell survival. YAP/TAZ signaling is involved in activation of hepatic stellate cells crucial in fibrogenesis. The availability of drugs (e.g. verteporfin) targeting the YAP/TAZ pathway are described as is their potential usage in veterinary medicine. The aim of this overview is to stimulate researchers’ and clinicians’ interest in the potential role of Hippo YAP/TAZ signaling in veterinary medicine.

## 1. Introduction

The ‘One Medicine’ concept exemplifies that differences between human and veterinary medicine hardly exist (Zinsstag et al. 2011). In line with this concept, it is conceivable that processes ranging from molecular pathways to surgical interventions, work *grosso modo* similar over various species, and as a consequence information derived in one particular species is of value for other species. For those reasons medical and veterinary schools are sometimes located in close proximity. However, the zoonotic risk (‘One Health’) often drives the two schools physically apart. Irrespective of these two diametrically opposing forces, it is generally accepted that where veterinarians and medical doctors meet the mutual benefits are obvious.

In line with the ‘One Medicine’ concept we review a highly conserved regulatory system, the Hippo YAP/TAZ signaling pathway, a pathway that sparked surprisingly little interest in veterinary medicine. The road-map outlined below is as follows: first a description of the pathway itself, second biological functions where YAP/TAZ seems crucial, third the few studies on YAP/TAZ in canine and feline medicine already published, and last some points for further research, both at cellular level and for potential direct clinical applications.

## 2. Hippo YAP/TAZ signaling

### 2.1. Homology

The Hippo pathway was first discovered in 1995 during a genetic screen for mutations that cause cellular overgrowth in fruit flies (*Drosophila melanogaster*) (Justice et al. 1995; Xu et al. 1995). It had a catalytic domain similar to the human myotonic dystrophy protein kinase discovered two years earlier (Mahadevan et al. 1993). In addition to *warts* (Justice et al. 1995) and *lats* (Xu et al. 1995), other players in this tumor suppressor pathway were elucidated including *Hippo* (*hpo*) (Wu et al. 2003; Harvey et al. 2003; Jia et al. 2003), *Salvador* (*sav*) (Tapon et al. 2002), *Mob as tumor suppressor* (*mats*) (Lai et al. 2005) and *Yorki* (*yki*) and *Scalloped* (*sd*) (Huang et al. 2005; Goulev et al. 2008; Koontz et al. 2013).

**Figure 1.**
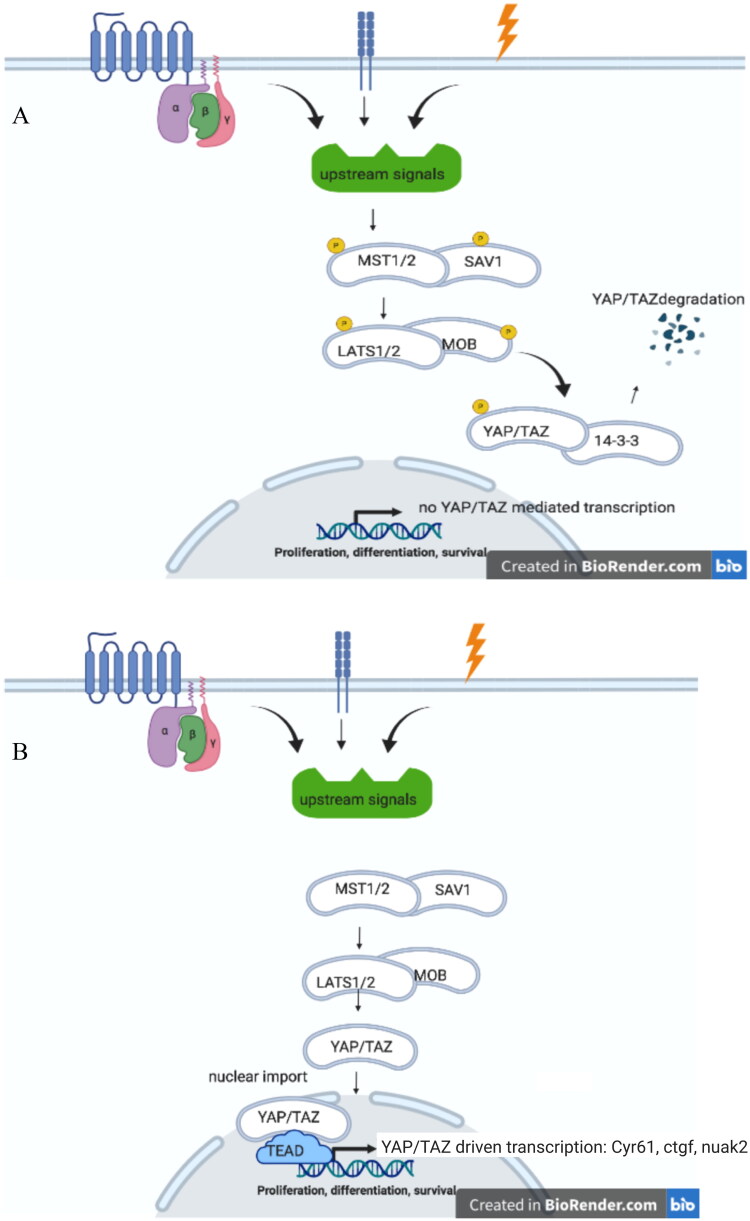
(A) Hippo On. Upon a plethora of external signals, such as G-protein coupled receptors, cell-adhesion, and (mechano)stress, MST1/2 (mammalian homologue of Drosphilia Hippo), and SAV1 are phosphorylated upon which LATS1/2-MOB protein become phosphorylated. This leads to phosphorylation of YAP/TAZ, that upon binding to 14-3-3 proteins degrades. (B) Hippo OFF. Due to lack of stimuli on MST1/2-SAV1, these protein remain unphosphorylated, as well as downstream proteins LATS1/2-MOB and YAP/TAZ. Unphosphorylated YAP/TAZ can enter the nucleus where, upon interaction ioth TEAD dries the transcription of specific gene products, such as *cyr61*, *ctgf* and *nuak2*.

The basic concept of the Hippo pathway in *Drosophila* are conserved in mammals (Figure 1). Mammalian STE20-LIKE PROTEIN KINASE 1/2 (MST1/2) is the mammalian homologue of Hippo (Chan et al. 2005; Callus et al. 2006), Salvador in named SALVADOR both in fruit flies and mammals, Warts and Mats are named LARGE TUMOR SUPPRESSOR KINASE 1/2 (LATS1/2) (Wu et al. 2003; Zhao et al. 2010) and MOB KINASE ACTIVATOR 1 (MOB1), respectively (Kim et al. 2016). Yorki in mammals consists of two proteins, viz. YAP (YES ASSOCIATED PROTEIN) and TAZ (TRANSCRIPTIONAL CO-ACTIVATOR WITH PDZ-BINDING MOTIF). In the nucleus YAP/TAZ interact with TEAD (TEA DOMAIN TRANSCRIPTION FACTOR) which is the mammalian homologue of Scalloped (Zhao et al. 2008). Active YAP/TAZ in conjunction with TEAD leads to enhanced transcription of several genes of which *ctgf*, *cyr61*, and *nuak2* (Connective tissue growth factor, and Cysteine-rich angiogenic inducer 61, nua kinase 2), are a few and these are often measured to indicate enhanced YAP/TAZ-activity. NUAK2 was recently described as a feed-forward activator of YAP/TAZ (Sun et al. 2013) implicated in liver cancer (Yuan et al, 2018).

How do all these components interact to elicit a transcription activation of specific target genes, and mediate proliferation and/or apoptosis? Upon stimulation via for instance G-protein coupled receptors or mechanosensing, phosphorylated scaffold protein SAV interacts with MST1/2 (Hippo) to allow phosphorylation of MST1/2 (Hippo). Phosphorylated MST1/2 (Hippo) initiates an interaction between scaffolding protein MOB (Mats) with LATS1/2 (Warts). This phosphorylated complex leads to a phosphorylated YAP/TAZ (Yorki) which is therewith inactivated (degraded via interaction with 14-3-3 protein) and not able to translocate into the nucleus. Active YAP/TAZ (unphosphorylated YAP/TAZ Yorki) mediates cell proliferation.

For reasons of readability from hereon we will refer to YAP/TAZ signaling indicating that the data are based on research in mammalian systems, and not solely dependent on the canonical Hippo kinase signaling.

### 2.2. Signaling cascade, from broad spectrum triggers to specific nuclear translocation and transcription activation

A plethora of extracellular signals modulate YAP/TAZ signaling. Upstream triggers of the YAP/TAZ signaling include for instance ligands for G-protein-coupled receptors like lysophosphatidic acid (LPA), sphingosine 1-phosphatese, and thrombin (that lead to YAP/TAZ activation), whereas glucagon, epinephrine, dopamine inhibit YAP/TAZ (reviewed in Yu et al. 2012a, 2012b). In view of YAP/TAZs role in organ size, and therefor cell-cell interactions it is not a surprise that tight junction and adherens junction protein in the plasma membrane inhibit YAP/TAZ-mediated proliferation as do signals initiated via focal adhesion (reviewed in Heng et al. (2020), and a beautiful graphical summary in Yu and Guan (2013)). A transmembrane protein called Crumbs (CRB1) is indispensable for establishing basolateral/apical cell polarity established via gap junctions, tight junctions and adherens junctions. The Crumbs polarity complex inhibits YAP/TAZ nuclear translocation to the nucleus by binding it, and as such established basolateral/apical polarity is maintained (Genevet and Tapon 2011). Lastly, stress fibers (F-actin) have been uncovered to guide the downstream effectors of the Hippo pathway (Wada et al. 2011; Seo and Kim 2018). Cells are stretched at low densities, and its morphology induces the formation of F-actin. F-actin inhibits Lats1/2 phosphorylation allowing YAP/TAZ to enter the nucleus. These are just a few examples of cytoskeletal components affecting YAP/TAZ signaling. For further details readers are referred to the reviews above and the original paper therein, as it is out-of-focus to go into great detail into the various initial inputs in the YAP/TAZ signaling (Yu and Guan 2013; Heng et al. 2020).

After various input signals are provided it converges to a linear sequence of events: MST1/2 becomes phosphorylated followed by phosphorylation of SAV, MOB, and LATS1/2. Phosphorylated LATS1/2 phosphorylates YAP/TAZ (on Ser127 and Ser89, respectively) which retains this complex form nuclear translocation. Cytoplasmic retention is mediated via cytoskeletal anchoring upon binding to 14-3-3 proteins (Basu et al. 2003). Furthermore, in its phosphorylated form YAP/TAZ is amendable for proteasomal degradation upon polyubiquitination (Zhao et al. 2010). When de-phosphorylated the YAP/TAZ complex enters the nucleus, and upon interaction with the transcription factor TEAD it initiates gene specific transcription (Figure 1).

The list of YAP/TAZ TEAD-mediated transcription includes at least 22 target genes of which *cyr61* (cysteine-rich angiogenesis inducer 61) is probably the most specific one (Wang et al. 2018). Not only was high YAP/TAZ correlated with poor prognosis in numerous cancers (Wang et al. 2018), it was also evident that the target genes were implicated in either one of the hallmarks of cancer (Hanahan and Weinberg 2011). These ten hallmarks are (1) evading growth suppressors; (2) avoiding immune destruction; (3) enabling replicative immortality; (4) tumor-promoting inflammation; (5) activating invasion and metastasis; (6) inducing angiogenesis; (7) genome instability and mutations; (8) resisting cell death; (9) deregulating cellular energetics; and (10) sustaining proliferative signaling (Hanahan and Weinberg 2011). For instance, DOCK5 (dedicator of cytokinesis5) and ARHGEF17 are involved in cell spreading and metastasis, whereas CYR61, AMOTL2 (angiomotin-like 2), ANKRD1 (ankyrin repeat domain-containing protein1), and CRIM1 (cystine-rich motor neuron protein 1) are implicated in angiogenesis. NUAK2, in a feed-forward loop with YAP/TAZ/TEAD plays a role in cellular energetics, and CTGF (an immediate-early gene induced by numerous growth factors), IGFBP3 (insulin growth factor binding protein 3), and PTN14 (protein tyrosine phosphatase nonreceptor-type 14) are associated with sustained proliferation signals. Resistance to cell death is, amongst other factors, mediated via ABCB1 (multidrug resistance pump) and TXN (thioredoxin).

### 2.3. YAP/TAZ upregulation as indictor for worse prognosis in cancer

In line with the YAP/TAZ target genes, upregulation of YAP/TAZ was associated with poor prognosis and/or poor clinical outcome in numerous cancers including lung, breast, colorectal, pancreatic, gastric, esophageal, bladder, skin, and gender specific tumors in the prostate, endometrium, and ovaria (Cordenonsi et al. 2011; Noguchi et al. 2014; Saadeldin et al. 2014; Moroishi et al. 2015; Bonilla et al. 2016; Lo Sardo et al. 2018; Sheng et al. 2019). The importance of YAP/TAZ in hepatocellularcarcinoma (HCC) is anticipated in view of the crucial role of YAP/TAZ in liver development (Lee et al. 2016), and the initiation of a ductular reaction (bile duct epithelial cell expansion) upon liver injury (Tschaharganeh et al. 2013; Yimlamia et al, 2014; Panciera et al. 2016; Planas-Paz et al, 2019; Pepe-Mooney et al. 2019). It is obvious that YAP/TAZ target gene product NUAK2 plays a crucial role in the YAP/TAZ-mediated liver tumor progression (Yuan et al. 2018). Numerous papers implicate aberrant YAP/TAZ signaling with HCC development *in vivo* (Corvaisier et al. 2016; Kim et al. 2016, 127; Kim et al. 2018; Zhang et al. 2018; Moon et al. 2019; Van Haele et al. 2019; Weiler et al. 2020; Bisso et al. 2020).

Taken together the importance of YAP/TAZ as potential therapeutic target and as prognosticator are obvious in human medicine. How is this in veterinary medicine?

## 3. Aberrant YAP/TAZ signaling in veterinary medicine

### 3.1. YAP/TAZ in canine and feline diseases

Surprisingly few papers are retrieved from a PubMed-search in Yap/TAZ with the addition of species like dog, cat, and horse (Beffagna et al. 2016; Rico et al. 2018; Luu et al. 2018). Actually, no single hit was found with horse or equine. First YAP/TAZ published studies in cats and dogs are presented followed by potential association of dysregulated YAP/TAZ in typical canine and feline liver pathologies.

The mammary gland tumor is the third most common neoplasm in queens (intact female cats) and the most common neoplasm in intact bitches. A descriptive study by Beffagna et al. from 2016 that examined mammary gland tumors in dogs and cats concluded that YAP/TAZ play a role in the development of those neoplasms (Beffagna et al. 2016). The study used an anti-WWRT1 rabbit antibody, which recognizes both YAP and TAZ, to do a semi-quantitative evaluation of positively stained cells. The results showed an increase of both phosphorylated cytoplasmic and non-phosphorylated nuclear TAZ in feline mammary tumors. The canine mammary tumors showed an increase as well but not as significantly as in cats. The diameter of the mammary gland tumors did not appear to be related to YAP/TAZ expression, because even in small tumors high levels of YAP/TAZ were found. The grade of the tumor did seem to correlate with YAP/TAZ expression as grade III tumors had a more significant increase of nuclear expression compared to grade I tumors. These results imply that YAP and TAZ not only play a role in tumorigenesis, but in aggressiveness, too. The same outcome has been established in studies in human breast cancer, suggesting similarity in development of human breast cancer and mammary gland tumors in canines and felines (Beffagna et al. 2016).

Another study on canine mammary gland tumors with a bigger cohort followed in 2018. In contrast to the study in mammary gland tumors discussed earlier, separate antibodies against YAP and TAZ were used so the different roles of both coactivators could be examined more closely. Rico et al. (2018) established that TAZ appears to play a bigger role in developing hyperplasia of mammary tissue or mammary gland tumors than YAP does. TAZ levels were elevated in mammary hyperplasia in both cytoplasm and nucleus, whereas in malignant mammary tumors TAZ was only significantly elevated in the nucleus. TAZ levels weren’t increased in either the cytoplasm or the nucleus in benign mammary tumors. This indicates an association between TAZ expression and both developing hyperplasia and acquiring a malignant phenotype. YAP expression was only significantly increased in malignant mammary tumors in the nucleus. This indicates that YAP expression can also be associated with developing malignancy. In this study YAP/TAZ levels did not differ between different grades of tumors like in the preliminary study of Beffagna et al. (2016). Possibly, the contrast between the studies could have been caused by the fact that different antibodies were used, and the tumor types examined might have been different (Beffagna et al. 2016; Rico et al. 2018).

On a related note, Luu et al. examined canine osteosarcoma *in vitro* and evaluated the involvement of YAP/TAZ crosstalk with TGF, with the use of YAP and TAZ specific small interfering RNAs to specifically reduced *yap* and *taz* expression (Luu et al. 2018). These siRNAs were used individually or in combination with pSmad2 silencing, a transcription factor indicating activity of TGF. The results of this study proved that YAP/TAZ signaling is indeed involved in the development of canine osteosarcoma. The results indicated that YAP and TAZ both tune metastasis-associated features of canine osteosarcoma. The study also concluded that YAP and pSmad2 could possibly be an interesting combination to look further into regarding prognosis of canine appendicular osteosarcoma (Luu et al. 2018; Portela et al. 2014). Unfortunately, this suggestion was not conceptualized.

To sum up the facts, *firstly*, studies have concluded that YAP/TAZ signaling plays a role in canine and feline diseases like in mammary tumors and osteosarcoma which means there is evidence that dysregulation of the pathway causes disease in dog and cat. *Secondly*, the pathway is highly conserved in mammals and there is proof that Hippo pathway dysregulation plays a part in diseases in liver disease specifically in mice and man. Those findings suggest that there is reason to believe that dysregulation of the pathway causes chronic liver disease in dog and cat as well as in man and mice. To conclude, according to the valid reasons above it is likely to believe that YAP/TAZ signaling is involved in canine and feline diseases in addition to the described role in mammary tumors and osteosarcomas.

### 3.2. Tools to study YAP/TAZ in veterinary medicine

In view of the evolutionary conservation of YAP/TAZ signaling and the clear indications that affected YAP/TAZ signaling is implicated in a wide variety of tumors one would expect that YAP/TAZ signaling would be investigated in-depth in veterinary medicine too. This, however, is obviously not the case. Just a limited number of papers describe YAP/TAZ in a veterinary setting (Beffagna et al. 2016; Rico et al. 2018; Luu et al. 2018). One of the reasons for this lack in data on disturbed YAP/TAZ signaling in veterinary pathologies probably lies in the fact that the analysis of this pathway is difficult. First activated YAP/TAZ can be measured at histological level by means of positive staining for either YAP or TAZ in the nucleus. This poses the investigators with problems associated with the specificity for antibodies for non-rodent animals, for which often data on specificity are lacking. This means that paraffin embedded tissues, although likely to be present in the various veterinary academia, is not ideally suited for measuring YAP/TAZ activation. Despite these difficulties YAP/TAZ activity was measured by means of immunohistochemistry in clinical samples (Beffagna et al. 2016; Rico et al. 2018). A more indirect approach could be the measurements of relative levels of target gene expression (Cao and Zhao 2019). This kind of assay requires affordable molecular equipment (PCR machines suitable for quantitative measurement and requires proper experimental design staring from the harvest of the material until the final calculation). For this the MIQE-guidelines are a user-friendly tools freely available (Bustin et al. 2010; Bustin and Penning 2012), as is a list of primers for numerous reference genes for dogs and cats (Brinkhof et al. 2006; Penning et al. 2007; Peters et al. 2007).

### 3.3. Clinical interference, specific inhibitors, possible therapeutic options

Since hepatic fibrosis is not easily treated and primary liver cancer is a very dangerous disease, a novel functioning treatment would be extremely helpful. Since the Hippo pathway is a key regulator of liver diseases such as hepatic fibrosis and liver neoplasia, it would be most convenient to target this cascade, as it seems to be the initiator in many cases. As noted previously, overexpression of either YAP or TAZ enhances the proliferation of liver cells, and the acquisition of a malignant phenotype. YAP and TAZ also augment the migratory capacity of the cells, indicating that YAP and TAZ play a role in metastasis of liver tumors (recent review by Thompson (2020)). When overexpression of YAP/TAZ is located in hepatic stellate cells it results in liver fibrosis followed by liver cirrhosis. So logically, the purpose of the treatment is to lower the expression of YAP/TAZ in selected liver cells by targeting the Hippo pathway.

Many drugs have been found to be able to alter YAP/TAZ expression in liver cells in order to inhibit liver fibrosis (Zhang et al. 2016; Ge et al. 2017; Zhang et al. 2018; Haak et al. 2019; Mohseni et al. 2019). One study even found a drug that may possibly reverse fibrosis. However, these drugs have neither been tested in dogs nor cats yet. These drugs are summarized in Table 1. In this paragraph the main focus will be on the one drug that has been examined in canine tissue: verteporfin (Visudyne®, Novartis Ophthalmics Europe Ltd, Hants, UK).

**Table 1.** Drug affecting YAP/TAZ-activation.

Drug name	Mechanism(s) of action	Effect(s)	Materials & Methods	References
**Verteporfin**	Increasing 14-3-3 proteins in the cytosol, trapping YAP in the cytosol and therefore inhibiting of association of YAP and TEAD in the nucleus	Suppression of cancer growth because of decreased CYR61 and CTGF levels	Human tissue *In vitro* canine mammary gland tissue	(Mohseni et al. 2019; Zhang et al. 2018) (Zhang et al. 2016)
**Carvacrol**	Reducing YAP/TAZ levels in the cell and inhibiting gene expression of TAZ	Decreased fibrosis development	*In vivo* rats with tetrachloride-induced liver fibrosis	(Mohseni et al. 2019)
**Oroxylin**	Modulating HIF-1alpha, suppression nuclear translocation of YAP	Inhibited tumor invasion and migration, induction of apoptosis and inhibition of angiogenesis in liver sinusoidal endothelial cells	*In vivo* male ICR mice with induced liver fibrosis	(Zhang et al. 2018)
**ω-3 PUFAs**	Promoting YAP/TAZ degradation	Inhibition of proliferation of hepatic stellate cells and activation of hepatic stellate cells	*In vivo* mice with tetrachloride-induced liver fibrosis	(Zhang et al. 2016)
**Dihydrotanshinone I**	Blocking nuclear translocation of YAP with significant CTGF downregulation	Stimulation of autophagic flux and promotion of degradation of liver collagen	*In vivo* rats subjected to bile duct ligation & in vitro human and rat hepatic stellate cell line	(Ge et al. 2017)
**Dihydrexidine** **(Dopamine agonist)**	Elevating cAMP through Dopamine receptors promoting YAP phosphorylation and downregulating CTGF	Reduction of extracellular matrix stiffness and cross-linking, contractile myofibroblast function and proliferation	*In vitro* hepatic stellate cells *In vivo* eight-week-old female and male C57BL/6 mice and transgenic mice	(Haak at al. 2019)

In 2012, Liu-Chittenden et al. examined the TEAD-YAP transcription factor complex as a possible therapeutic target (Liu-Chittenden et al. 2012). They looked into small molecules from the porphyrin family that could possibly inhibit the physical association between TEAD and YAP, making gene expression impossible. The compounds examined were protoporphyrin IX, hematoporphyrin and verteporfin. The results of the study show that protoporphyrin IX and verteporfin significantly inhibit YAP-TEAD interaction, with verteporfin being the more powerful inhibitor. This inhibition of YAP-TEAD association resulted in an arrest of liver overgrowth and therefore inhibition of YAP-induced tumor growth in the liver (Liu-Chittenden et al. 2012). Another study found out how verteporfin causes a disruption in the YAP-TEAD association. The results demonstrated that verteporfin causes an increase in 14-3-3 proteins in the cytosol, leading to YAP trapping in the cytosol and therefore inhibition of association of YAP and TEAD in the nucleus. YAP/TAZ target gene expression is inhibited meaning cancer growth will be suppressed (Wang et al. 2016).

The studies described above were about human liver tissue. Verteporfin has also been proven to be effective in canine cancer, though in mammary gland tissue instead of liver tissue. The results of the study in canine mammary tumors indicate that verteporfin sensitivity is associated with YAP expression in the cell, suggesting that the cytotoxic effect of the molecule is dependent on YAP expression. The cytotoxic effect of verteporfin relies on induction of apoptosis and doesn’t have a significant effect on cell proliferation of tumor cells. They also examined the effect of verteporfin on expression of YAP/TAZ target genes. Measuring mRNA levels of YAP/TAZ genes relevant in cancer (CTGF and CYR61) after treatment with verteporfin yielded that both CTGF and CYR61 levels decreased remarkably. Furthermore, scratch assays, a classical method to investigate migration/invasion/metastasis showed that verteporfin inhibited these cell processes. They also discovered that verteporfin inhibits anchorage-independent growth in canine mammary gland tumor cell lines CMT-28 and CMT-47 (Guillemette et al. 2017). Together these data indicate a potential for verteporfin in canine mammary gland tumor treatment. Of critical note here is that verteporfin does cause photosensitivity, a feature that needs to be taken into account *in vivo*. The latest tool to inhibit YAP/TAZ is based on NUAK2 inhibition, and this drug works in the nanomolar range, yet its tolerance for dogs and cats has not been established yet (Yuan et al, 2018).

In a pre-press paper by Sammarco et al a comparison of human, canine and feline mammary tumors was made including mRNA levels of YAP/TAZ and downstream target genes like CTGF (Sammarco et al. 2020). This paper included primer sequence to analyse these gene products in man, dog and cat. Of interest whereas relative mRNA levels were generally comparable between healthy and tumor tissue, at the protein levels a clear difference was observed, with increased nuclear TAP/TAZ in higher graded mammary tumor tissue of cat and dog (Sammarco et al. 2020).

## 4. Future clinical perspectives

The results of these studies show important points for clinical practice: (1) based on the conservation of this pathway and the ‘One Medicine concept’ it is highly conceivable that YAP/TAZ signaling is affected in various pathologies relevant for veterinary medicine, especially in the fields of oncology and hepatoloy; (2) that there are a few possible drugs that could target the YAP/TAZ pathway. Neither measurements of YAP/TAZ signaling are fully exploited to come to mechanistical answers and as a potential factor for disease prognosis, nor are the currently available drugs being tested in companion animals. This review is an invitation to join forces to investigate YAP/TAZ to the benefit of animals. The hippopotamus is the mammal (next to men) responsible for the most killings in Africa, together we can make sure that Hippo YAP/TAZ will not be a big killer for our companion animals.
